# Development of omega‐3‐rich *Camelina sativa* seed oil emulsions

**DOI:** 10.1002/fsn3.572

**Published:** 2017-12-21

**Authors:** Henok D. Belayneh, Randy L. Wehling, Yue Zhang, Ozan N. Ciftci

**Affiliations:** ^1^ Department of Food Science and Technology University of Nebraska‐Lincoln Lincoln NE USA

**Keywords:** camelina seed oil, emulsion, homogenization, oxidation, stability

## Abstract

*Camelina sativa* seed is an underutilized oil source rich in omega‐3 fatty acids; however, camelina oil is not fully explored for food applications. Its high omega‐3 content makes it susceptible to oxidation, which may limit food applications. Therefore, the main objective of this study was to investigate the potential of camelina seed oil to form physically and oxidatively stable emulsions as a potential delivery system for omega‐3 fatty acids. Effects of homogenization conditions, namely, pressure (15 MPa‐30 MPa), number of passes (1,3,5, and 7), and type of homogenizers (high pressure and high shear) on the structural properties and stability of camelina seed oil emulsions stabilized with whey protein isolate were studied. High homogenization pressure (30 MPa) and number of passes (>3) reduced the particle size (278 nm) and formed more physically and oxidatively stable emulsions compared to high shear homogenization; high shear homogenization generated bigger oil particles (~2,517 nm). Apparent viscosity and consistency index (k) decreased with increasing pressure, number of passes, and shear rate. Emulsions prepared with high pressure homogenization at both 15 and 30 MPa with 3 and more passes did not exhibit any peroxide formation over 28 days. Results indicated that camelina seed oil is a promising alternative oil source to form stable omega‐3‐rich emulsions for food applications.

## 1 INTRODUCTION

Camelina seed (*Camelian sativa* L. crantz) is a little‐known oil seed which belongs to the *Brassicaceae* family (Mansour et al., 2014; Shukla, Dutta, & Artz, 2002). Even though the demand for camelina seed in the United States has been for biodiesel production because of its high oil (28%–40%) (Budin, Breene, & Putnam, 1995; Li, Qi, Sun, & Wang, 2016; Lu, Napier, Clemente, & Cahoon, 2011; Nguyen et al., 2013), its high omega‐3 and minor lipid components such as tocopherols and phytosterols make it suitable for food applications. Camelina seed oil has very recently received a Generally Recognized As Safe (GRAS) status by Food and Drug Administration (FDA) of the United States (GRN642, 2016). Camelina seed oil contains 33%–40% of α‐linolenic acid (C18:3 ω3, ALA), an essential omega‐3 fatty acid (Belayneh, Wehling, Cahoon, & Ciftci, 2015) known for health benefits such as promoting eye health and development of brain and nervous system in infants, reducing risk of hypertension, and some types of cancer (Chiu, Klein, Milton, Gensler, & Taylor, 2009; Gogus and Smith 2010). In addition, camelina seed oil is rich in tocopherols (760 mg/kg oil) and phytosterols (up to 6,500 mg/kg oil) which are natural antioxidants and health promoting minor lipid compounds (Schwartz, Ollilainen, Piironen, & Lampi, 2008; Belayneh et al., 2015).

To date, marine oils have been the main sources of omega‐3 fatty acids, however, sustainability of these sources is a challenge, they are not shelf‐stable, not palatable to many consumers, not easily accessible, and relatively more difficult to process and handle. Therefore, there is an increased need for alternative more sustainable sources of omega‐3 fatty acids. Production of sustainable oil crops and mainly re‐evaluation of underutilized oil seeds such as camelina seed has thus been considered as a new source for omega‐3 oils (Adarme‐Vega, Thomas‐Hall, & Schenk, 2014).

However, high PUFA content of camelina seed oil makes it susceptible to oxidation. Emulsion formation has been used as a method to improve the stability of oxidation‐sensitive oils while delivering PUFA. Emulsions also can mask uncommon smell of food ingredients such as camelina seed oil where its odor is strong to new consumers (Durgin & Hanan, 2004), and offer better organoleptic properties than oily solutions or forms that are administered orally (Kilcast, 2003). Couedelo et al. (2011) reported that there is a better bioavailability of the omega‐3 fatty acid, ALA, when flaxseed oil is consumed in the form of an emulsion. Kaci et al. (2016) also reported that emulsions can improve delivery of hydrophobic bioactive compounds such as coenzyme Q_10_. In addition, since emulsions consist of smaller particles, they promote a better gastro‐intestinal absorption of omega‐3 fatty acids (Remington & Beringer, 2005).

Therefore, the objective of this study was to develop camelina seed oil emulsions as an alternative omega‐3 delivery system, and to determine the effect of homogenization technique and homogenization conditions on the properties and oxidative stability of camelina seed oil‐in‐water (O/W) emulsions.

## 2 MATERIAL AND METHODS

### 2.1 Material

Certified organic camelina seed was purchased from a local market in Seattle, WA, USA, and its oil was extracted by a screw press (AgoilPress, M70 Oil Press Eau Clair, Wisconsin, USA). Whey protein isolate (WPI) was obtained from a local super market in Lincoln, NE, USA. Ammonium thiocyanate and barium chloride were purchased from Fisher scientific (Fair Lawn, NJ, USA). Ferrous sulfate heptahydrate was obtained from MP Biochemicals, LLC (Solon, OH, USA). All other reagents and solvents were of analytical or chromatographic grade and purchased from Sigma Aldrich (St. Louis, MO, USA).

### 2.2 Preparation of emulsions

Before forming the emulsions, WPI stock solution (5%, w/v) was freshly prepared by dissolving WPI in deionized water (pH 6.7) by stirring for 90 min at room temperature. After thorough dissolution, the pH of the solution was adjusted to 7.0 ± 0.2 using 2.0 mol/L NaOH. The solution was kept in a water bath (HAAKE N3, Thermo Scientific, Waltham, MA, USA) overnight at 10°C to ensure complete dissolution of the WPI. Once the stock solution was prepared, 3%‐WPI‐solution(w/v) was prepared by diluting the WPI stock solution with deionized water. Then, camelina oil and the 3%‐WPI‐solution (w/v) was mixed to obtain a final mixture with 30% (v/v) camelina oil concentration. The whole solution was premixed using an overhead mixer to facilitate mixing, and then homogenized. Two types of homogenizers, namely, high pressure (HC 5000, Microfluidics Corp., Newton, MA) and high shear, (Ultra‐Turrax T25, IKA Works, Inc., Wilmington, NC, USA) were used to form the emulsions. High‐pressure homogenizations were carried out at different pressures (15 and 30 MPa) and number of passes (1, 3, 5, and 7). Another set of emulsions were prepared using the high shear homogenizer at 10,000, 15,000, and 20,000 rpm (1400, 3150 and 5600 x g) for 10 min.

### 2.3 Creaming stability

Physical stability of the camelina seed oil emulsions was studied in terms of creaming stability according to Kuhn and Cunha (2012). Once the emulsions were prepared, 8 ml of sample from each emulsion was transferred into cylindrical glass tubes with plastic caps (internal diameter = 11 mm, height = 94 mm) and stored at room temperature for 28 days. Phase separation was followed by measuring the height of the emulsion (H_*T*_) and height of the transparent serum layer (H_*S*_) on the 2nd and 28th day. The extent of creaming was expressed as creaming index or stability (%, creaming stability) using the following equation:

(1) $$\mathrm{Creaming}\;\mathrm{index}\;\;\left(\mathrm{stability}\right)\left(\;\%\right)=\frac{H_{s}}{H_{T}}\times 100$$

### 2.4 Microstructure

An optical microscope equipped with a camera (AmScope, Irvine, CA) was used to visualize microscopic images that represent the microstructure of all emulsions 24 hr after their preparation. Samples were added onto microscope slides and covered with glass cover slides and observed using ×100 objective lenses at room temperature.

### 2.5 Particle size distribution and zeta potential

Oil droplet size and zeta potential of all emulsions were analyzed using a Zetasizer (Nano series 6.3, Malvern Instruments Ltd., UK) 2 days after their preparation. One ml of emulsion was added to 99 ml of distilled water at room temperature (20°C) and mixed thoroughly. The uniform dispersion was transferred into plastic cuvettes and capillary cells for measurement of size and zeta potential, respectively. Particle size was given as Z‐average diameter (nm) and the particle size distribution curves were expressed as percent intensity vs. diameter (nm). Zeta (ζ) potential was expressed in mV. Mean diameter (d_4,3_) was calculated as in Eq. (2).

(2) $$d_{43}=\frac{\sum n_{i}d_{i}^{4}}{\sum n_{i}d_{i}^{3}}$$

where *n*_*i*_ represents the number of oil droplets with a diameter of *d*_*i*_.

### 2.6 Rheological measurements

Shear stress, shear rate, and viscosity of the emulsions were studied using a Physica MCR301 modular rheometer (Anton Paar, Houston, TX, USA) equipped with a stainless steel parallel plate geometry (50 mm) and a zero‐gap set to 0.7 mm. All emulsions were studied one day after their preparation at 25°C and their flow curves were obtained using up‐down‐up steps after applying shear rates ranging from 0 to 300 s^−1^. Flow behaviors of the emulsions were modeled using Power law (Ostwald de Waele) which relates the shear stress τ (Pa) with the shear rate γ (s^−1^) over the ranges covered as given in Eq. 3.

(3) $$\tau =k.\gamma^{n}$$

where τ represents shear stress (Pa), *k* is consistency index (Pa.S^n^), γ is the shear rate (s^−1^), and *n* is the dimensionless flow behavior index.

### 2.7 Oxidative stability

Oxidative stability of the camelina seed oil emulsions was evaluated with an accelerated oxidation test at 50°C over 45 days of storage by following peroxide formation. Peroxide value (PV) was determined colorimetrically using an Evolution 201 UV‐Vis spectrophotometer (Thermo Fisher Scientific Inc., Waltham, MA, USA) according to Shantha and Decker (1994) with some modifications. First, oil was extracted from the emulsions by adding about 0.3 ml of emulsion to 1.5 ml of isooctane: isopropanol (3:2, v/v). The mixture was mixed by vortexing for 10 sec and then centrifuged at 3,400 *g* for 10 min (Allegra X‐15R Centrifuge (Beckman Coulter Inc., Brea, CA, USA) at 23°C. Then, the clear upper solvent layer (0.2 ml) was removed and mixed with 7.8 ml of chloroform:methanol solution (7:3, v/v) in a separate tube and vortexed again. Ammonium thiocyanate solution (30 μl) and iron (II) solution (30 μl) were added to the mixture and vortexed for 10 sec. The mixture was then incubated in a dark place for 5 min at room temperature and the absorbance was measured at 505 nm against a blank that contained all the reagents except the sample. A standard curve of cumene hydroperoxide was prepared to determine the hydroperoxide concentration. The peroxide value was expressed as milliequivalents of peroxide per kilogram of emulsion.

### 2.8 Statistical analysis

Data were presented as mean ± standard deviation (SD) based on triplicate experiments. Statistical analyses of the data were carried out by ANOVA and least‐squares difference (LSD) using SAS (version 9.4, SAS Institute Inc., Cary, NC, USA) software package at 95% confidence interval.

## 3 RESULTS AND DISCUSSION

### 3.1 Creaming stability

**Figure 1:**
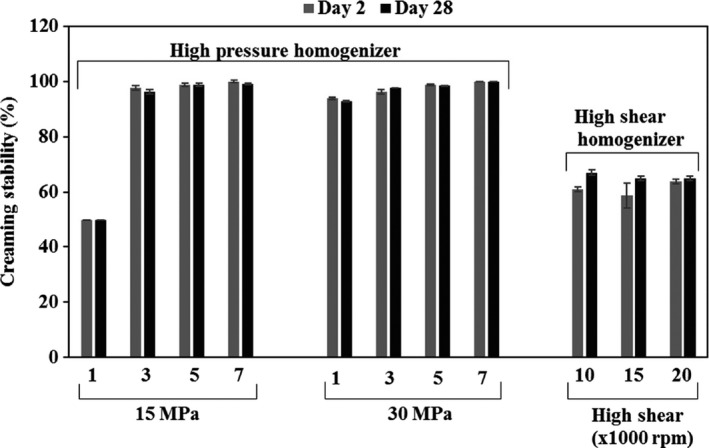
Effect of homogenization method and condition on the creaming stability of the camelina seed oil emulsions

**Figure 2:**
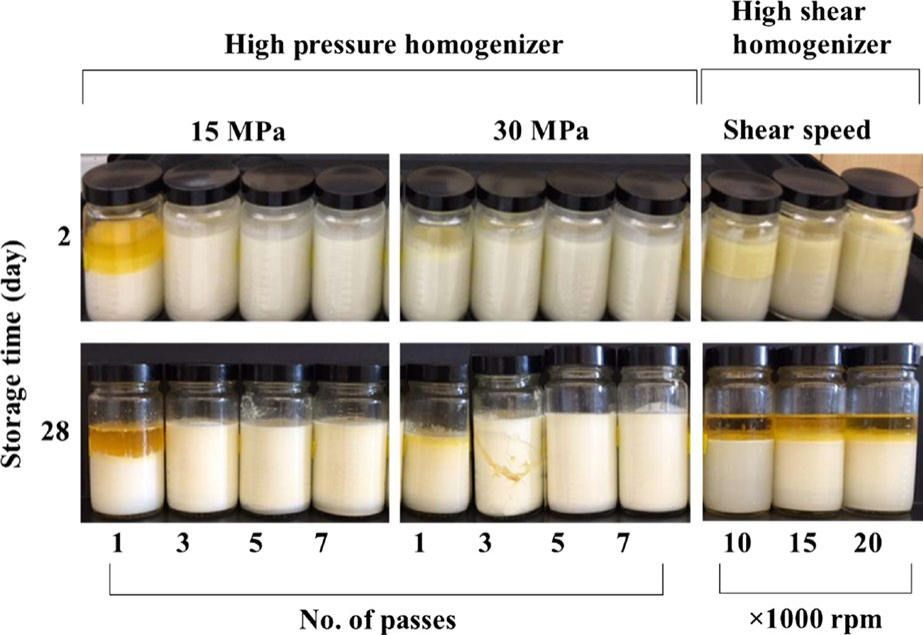
Visual appearance of camelina seed oil emulsions prepared using high‐pressure homogenizer and high shear homogenizer on day 2 and day 28

Figure 1 presents creaming stability of the camelina seed emulsions prepared at different conditions. High‐pressure homogenization generated more stable emulsions compared to high shear homogenization, except for 1 pass high‐pressure homogenization at lower pressure (15 MPa). Regardless of the number of passes, emulsions prepared with high‐pressure homogenizer at 30 MPa resulted in the highest creaming stability. Increasing number of passes from 1 to 3 at both 15 and 30 MPa increased the creaming stability significantly from 49.4% and 93% to 97.4% and 97.6%, respectively (*p* < .05); however, further increase in the number of passes did not cause a significant increase in the creaming stability (*p* > .05). This behavior was attributed to the coalescence of the fat droplets as a result of high attrition and temperature buildup at higher pressures and more than three passes. The emulsions prepared using high shear homogenizer demonstrated significant creaming (Figure 2) and their creaming stability ranged from 58.8% to 67.1% (Figure 1). There was no significant difference between the creaming stability of the emulsions prepared at different shear rates. When the creaming stabilities at 2nd and 28th days were compared, there was no significant difference between the stabilities of the emulsions prepared using high‐pressure homogenizer (*p* > .05), whereas, the phase separation in the emulsions prepared by high shear homogenizer at 28th day was significantly higher than those at 2nd day. Camelina oil emulsions prepared using the high‐pressure homogenizer at 30 MPa with 5 and 7 passes appeared to be physically stable for longer period (more than 2 months) with no clear phase separation compared to emulsions reported in other studies for flaxseed oil emulsions (Kuhn and Cunha 2012; Goyal et al., 2015). Kuhn and Cunha (2012) reported a WPI‐stabilized flaxseed oil emulsion which was physically stable for about 1 month. Hebishy and others (2015) studied sunflower and olive oil emulsions stabilized with WPI and produced by ultrahigh‐pressure homogenization, and reported that phase separation was observed after about 3 weeks for the most stable emulsion. The higher physical stability of WPI‐stabilized camelina seed oil emulsion was attributed to the phospholipids available in the camelina seed oil; major phospholipid in camelina seed oil is phosphatidylinositol (Belayneh, Wehling, Cahoon, & Ciftci, 2017). The results suggested that phosphatidylinositol‐rich phospholipid originally present in the oil synergistically improved the stability of the emulsions. Previously, synergistic effect of two emulsifiers, namely, lecithin and glycerol monostearate, on improving emulsion stability was reported by Moran‐Valero, Ruiz‐Henestrosa, and Pilosof (2017).

### 3.2 Microstructure and particle size distribution

**Figure 3:**
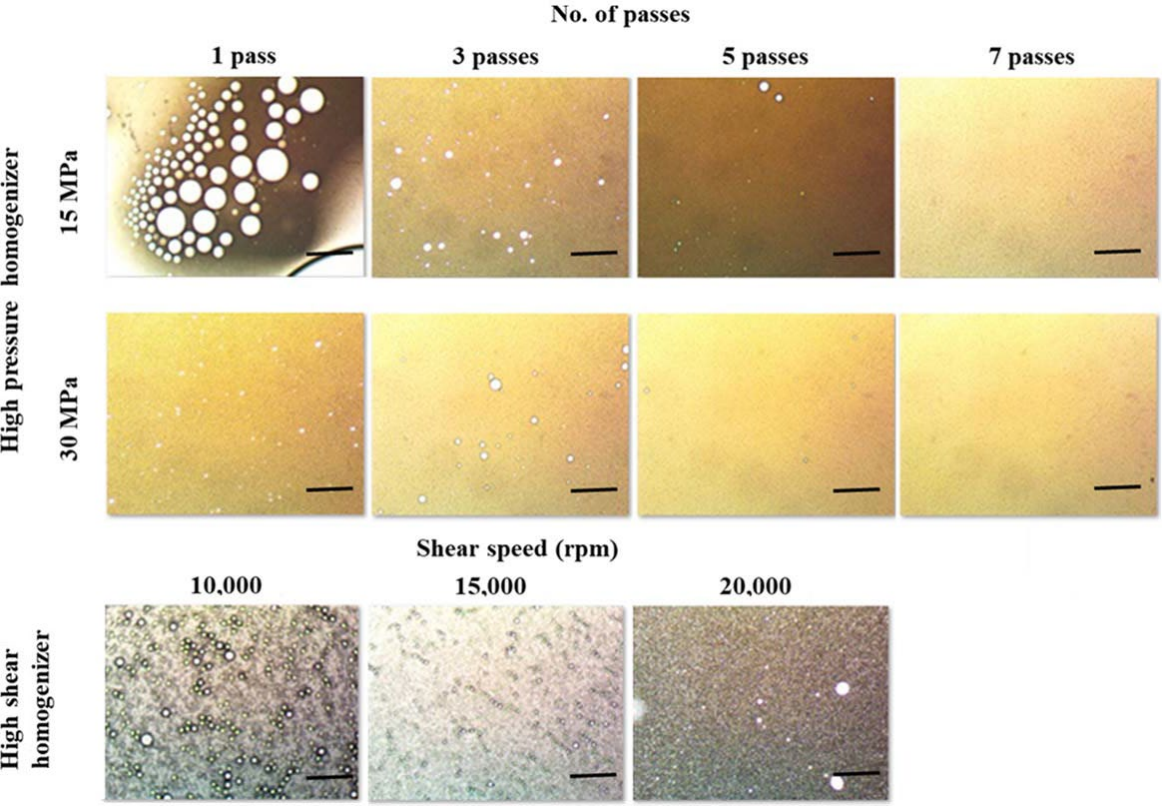
Effect of homogenization method and condition on the microstructure of the camelina seed oil emulsions

Optical microscope images of the emulsions prepared using both high‐pressure homogenization and high shear homogenization are shown in Figure 3. Increasing pressure decreased the oil droplet size, and this was more significant at homogenization with 1 pass. Increasing number of passes at the same pressure also decreased the oil droplet size and formed a more uniform oil distribution. Previously, Kuhn and Cunha (2012) and Qian and McClements (2011) reported similar observations when they studied effect of pressure on flaxseed oil emulsions and nanoemulsions stabilized by model food grade emulsifiers, respectively. Camelina seed oil emulsions obtained by high shear homogenizer contained bigger oil droplets compared to the ones obtained by high‐pressure homogenization, because high‐pressure homogenizers create intense turbulence, shear, and cavitation that disrupts and breaks down the fat droplets unlike the high shear homogenizers (McClements, 2011). Homogenizing the emulsions using high shear homogenizer at 10,000 rpm for 10 min resulted in coalescence of the fat droplets, whereas 20,000 rpm resulted in a more homogeneous oil droplet distribution (Figure 3).

**Figure 4:**
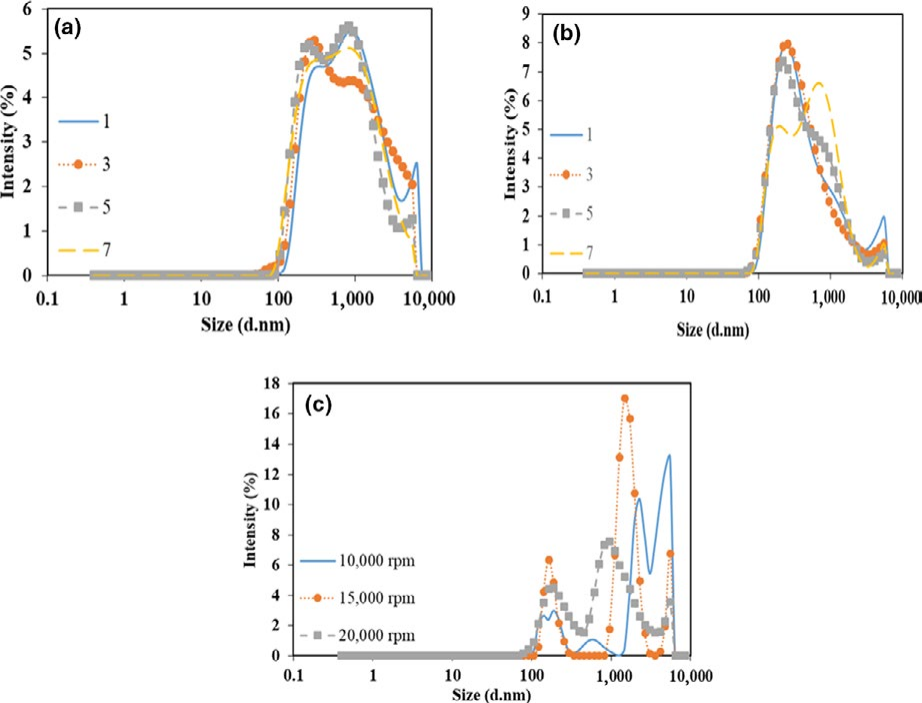
Particle size distribution of the camelina seed oil emulsions homogenized at (a) 15 MPa, (b) 30 MPa, and (c) different shear rates

Particle size distribution of the camelina seed oil emulsions are shown in Figure 4. The use of high‐pressure homogenizer resulted in emulsions with a narrower and more uniform particle size distribution (Figure 4a and b). Even though the particle size range (~100‐8,000 nm) was similar for the emulsions prepared using high‐pressure homogenizer at both 15 and 30 MPa, emulsions prepared at 30 MPa contained higher amount of smaller oil droplets. Increasing the homogenization pressure from 15 to 30 MPa resulted in a narrower particle size distribution. Homogenization at 30 MPa with 7 passes broadened the oil droplet size range and formed a biomodal size distribution by shifting the major peak from 300 to 100 nm. This behavior was due to coalescence caused by increased cavitation. A similar observation was reported by Kuhn and Cunha (2012) where increasing the pressure to 80 MPa and the number of passes above 3 in WPI stabilized flaxseed oil emulsion resulted in coalescence and thus bigger particle sizes. On the other hand, homogenization at 15 MPa formed emulsions with significant number of oil droplets with particle sizes greater than 1,000 nm. The particle size distribution of the emulsions prepared using high shear homogenizer was trimodal at 15,000 and 20,000 rpm, whereas lower shear speed (10,000 rpm) homogenization generated bigger droplets with a more nonuniform size distribution (Figure 4c). However, the particle size range of the emulsions obtained with high‐pressure homogenization and high shear homogenization did not differ.

Average oil droplet size ranged from 462 (7 passes) to 556 nm (1 pass) in the camelina seed oil emulsions prepared at 15 MPa using high‐pressure homogenization, and this range was between 278 (3 passes) and 394 nm (7 passes) when the homogenization pressure was 30 MPa (Table 1). At 30 MPa, 3 and 5 passes resulted in the most significant decrease in average particle size (*p* < .05). The lowest average particle size (278 nm) was obtained at 30 MPa homogenization with 3 passes. Increasing the number of passes to 5 and 7 at 30 MPa led to an increase in average particle size, suggesting aggregation of the oil droplets, which is also seen in Figure 4. When the emulsions were prepared using high shear homogenizer at 10,000 and 15,000 rpm, the average particle size was more than eightfold of the average size obtained using the high‐pressure homogenizer. However, when the shear speed was increased to 20,000 rpm, the average particle size of the emulsion decreased significantly to 735 nm (*p* < .05). The results agree with the observations reported by Goyal et al. (2015) and Kunh and Cunha (2012) for flaxseed oil emulsion stabilized using different emulsifiers. Kunh and Cunha (2012) observed particle sizes as low as 460 nm when the pressure was increased to 80 MPa with 4 number of passes.

**Table 1:** Particle size distribution, zeta (ζ)‐potential, and polydispersity index (PDI) of camelina seed oil emulsions

	High‐pressure homogenization								High shear homogenization		
	15 MPa				30 MPa				Shear speed (×1,000 rpm)		
	Number of passes								10	15	20
	1	3	5	7	1	3	5	7			
Z‐average (d.nm)	556 ± 21	536 ± 12	532 ± 94	462 ± 67	363 ± 32	278 ± 40	362 ± 57	394 ± 20	2517 ± 71	2624 ± 260	735 ± 43
Zeta potential (mV)	−36.8 ± 18.4	−48.7 ± 4.5	−54.1 ± 0.2	−50.8 ± 1.9	−51.8 ± 5.1	−53.1 ± 3.5	−54.4 ± 1.1	−54.7 ± 1.2	−52.5 ± 3.1	−49.9 ± 2.6	−50.6 ± 5.4
Polydispersity index (PDI)	0.59 ± 0.19	0.49 ± 0.00	0.42 ± 0.03	0.43 ± 0.05	0.40 ± 0.00	0.38 ± 0.05	0.39 ± 0.05	0.43 ± 0.01	0.43 ± 0.02	0.93 ± 0.01	0.66 ± 0.03

Zeta potential (mV) and polydispersity index (PDI) of all the camelina seed oil emulsions are also given in Table 1. Zeta potential, which is a measure of the electrostatic force on droplet surface, affects rheological properties of emulsions (Ozturk, Argin, Ozilgen, & McClements, 2015). Higher magnitude of zeta potential indicates higher stability of emulsions (McClements, 2011). WPI is negatively charged at neutral pH and thus the zeta potential of all emulsions was negative (Table 1). Emulsions prepared using high‐pressure homogenizer at 30 MPa showed the highest zeta potential values ranging between −51.8 and −54.7 mV. The lowest value was observed for the emulsion prepared using high‐pressure homogenizer at 15 MPa with 1 pass and this can be attributed to the fact that the pressure and number of passes were not enough to significantly lower the particle size and cover the droplets with the WPI. Similar results were reported by Nikovska (2012) and Saglam, Venema, Vries, Shi, and Linden (2013) who studied olive oil emulsions stabilized with soy protein isolate and WPI, and WPI, gum Arabic and Na‐caseinate stabilized sunflower oil emulsions, respectively. Emulsions with the highest zeta potentials were tended to have smaller particle sizes and be more stable. The relatively higher zeta potential values for most of the emulsions suggested that the amount of WPI used as an emulsifier was enough and efficient leading to a reasonably stable emulsion. The emulsion formed using the high‐pressure homogenizers at 15 MPa with 1 pass was the least stable based on its relatively lower zeta potential (−36.8 mV). Lower PDI results (0.38–0.43) showed that the emulsions prepared at 30 MPa homogenization were more homogeneous compared to the emulsions prepared at 15 MPa where the PDI ranged between 0.42 and 0.59 (Table 1). PDI results support the more uniform particle size distributions observed in Figure 4. However, emulsions prepared using high shear homogenizer at 15,000 and 12,000 rpm tended to be more heterogeneous with PDI values between 0.93 and 0.66, respectively. Even though the emulsions prepared at 10,000 rpm shear speed had lower PDI (0.43), they had greater particle size and lower creaming stability.

### 3.3 Rheological properties

**Figure 5:**
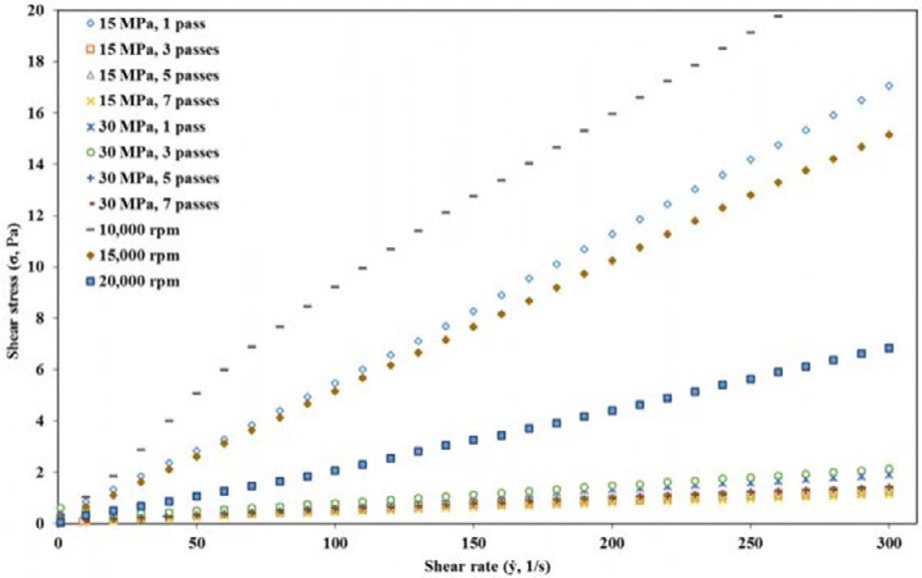
Shear stress (σ) of camelina seed oil emulsions prepared using high‐pressure homogenizer and high shear homogenizer as a function of shear rate (ẏ)

The flow curves in the form of shear rate versus shear stress for all camelina seed oil emulsions are presented in Figure 5. Increasing shear rate resulted in a linear increase in the shear stress in all emulsion samples. However, the response was more pronounced in the emulsions prepared using the high shear homogenizer compared to the ones prepared by high‐pressure homogenization. The highest shear stress was recorded in the emulsion prepared using high shear homogenizer at 10,000 rpm followed by the emulsion prepared using the high‐pressure homogenizer at 15 MPa with 1 pass. Other emulsions that showed a remarkable increase in their shear stress with increasing shear rate were the emulsions prepared at 15,000 and 20,000 rpm. The emulsions that were found to be physically stable tended to exhibit a lower shear stress with increasing shear rate.

**Figure 6:**
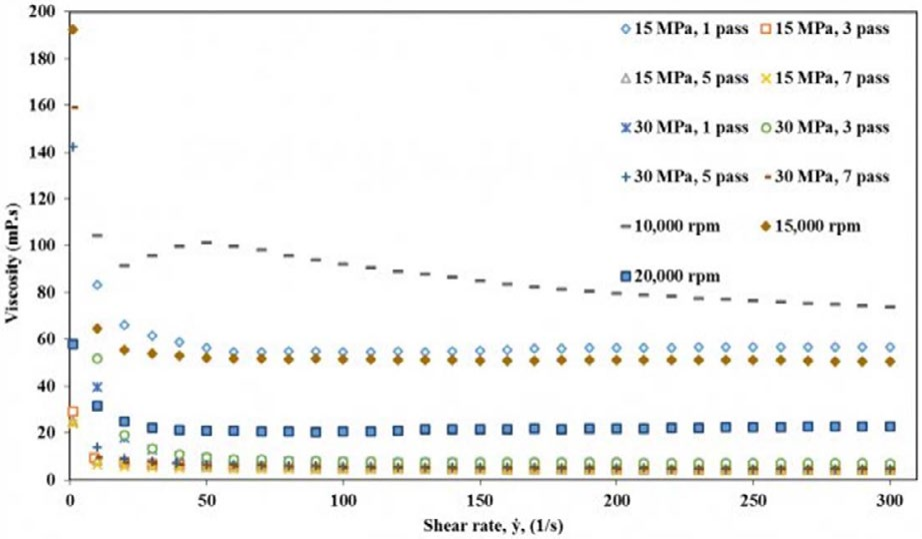
Apparent viscosity (η) of camelina seed oil emulsions prepared using high‐pressure homogenizer and high shear homogenizer as a function of shear rate (ẏ)

Figure 6 presents the change in the apparent viscosity of the camelina seed oil emulsions with shear rate (0–300 s^−1^) studied 1 day after their preparation. All emulsions showed a non‐Newtonian (shear thinning) behavior as evidenced by the decrease in the viscosity with increasing shear rate due to irreversible deformation and breakdown of the flocs under the shear stress (Kuhn and Cunha 2012). The viscosity was higher for all the emulsions at lower shear rates (0–20 s^−1^). Previously, high viscosities at low shear rates were reported by Zinoviadou, Scholten, Moschakis, and Biliaderis (2012) for emulsions prepared using WPI as emulsifier. Shear thinning is the most common flow behavior in foods and similar results were observed for emulsions prepared using different techniques (Kuhn and Cunha 2012; Goyal et al., 2015). Viscosities were higher for the emulsions prepared by high shear homogenizer compared to the emulsions prepared by high‐pressure homogenizer. This was more evident with increasing shear rate. Like the shear stress, the apparent viscosity was highest for the less physically stable emulsions followed by the emulsion prepared using the high‐pressure homogenizer at 15 MPa with 1 pass. This was attributed to the presence of bigger fat droplets in the emulsions prepared using the high‐pressure homogenizer at 15 MPa with 1 pass and all the emulsions prepared using high shear homogenization. The flow curve data for all of the WPI‐stabilized camelina seed oil emulsions were fitted to Power law model. High *R* ^2^ (>.921) for all of the emulsions suggested that the model could explain the relationship between the shear rate and shear stress (Table 2). Flow behavior index (*n*) obtained from the model indicated that the emulsions had low pseudoplastic behavior in agreement with the observations made from the flow curves. Except for the emulsion prepared using the high shear homogenizer at 15,000 rpm, the flow index for the emulsions varied from 0.749 to 0.990 and are thus considered as shear thinning. Kuhn and Cunha (2012) reported similar observations for flaxseed oil emulsions stabilized by WPI. In contrary, Goyal et al. (2015) observed higher pseudoplastcity in flaxseed oil emulsions prepared using different emulsifiers. Consistency index (k), a parameter related to the apparent viscosity, was higher for the emulsions prepared with 1 pass at both 15 and 30 MPa using high‐pressure homogenizer, whereas increasing the number of passes decreased consistency index at both pressures. The apparent viscosity at 100 s^−1^, which is a shear rate commonly encountered in food processes, increased with increasing pressure and number of passes, except for the emulsions prepared with 1 pass (Table 2). High shear homogenizers resulted in higher apparent viscosities and consistency index in agreement with the observations made in Figures 5 and 6. The consistency index (k) ranged from 0.011 for the camelina seed oil emulsion prepared at 30 MPa and 7 passes to 0.223 Pa.S^n^ for the ones prepared at 10,000 rpm. This suggests that camelina seed oil emulsions prepared using the high‐pressure homogenizers at higher pressures and number of passes were characterized by lower viscosity and smaller oil droplet size.

**Table 2:** Rheological parameters from the power law model and apparent viscosity at 100 s^−1^ for the camelina seed oil emulsions

	High‐pressure homogenization								High shear homogenization		
	15 MPa				30 MPa				Shear speed (×1,000 rpm)		
	Number of passes								10	15	20
	1	3	5	7	1	3	5	7			
*n*	0.990 ± 0.032	0.771 ± 0.032	0.817 ± 0.012	0.791 ± 0.042	0.749 ± 0.022	0.854 ± 0.030	0.818 ± 0.023	0.848 ± 0.013	0.796 ± 0.053	1.051 ± 0.120	0.990 ± 0.071
K (Pa.S^n^)	0.051 ± 0.002	0.014 ± 0.002	0.011 ± 0.001	0.013 ± 0.003	0.042 ± 0.019	0.015 ± 0.004	0.012 ± 0.003	0.011 ± 0.000	0.223 ± 0.094	0.041 ± 0.030	0.017 ± 0.002
η_100_ (mPa.S)	48.9 ± 5.3	4.8 ± 0.7	4.6 ± 0.4	4.9 ± 0.3	12.2 ± 4.4	7.6 ± 2.2	5.1 ± 0.7	5.4 ± 0.2	84.8 ± 18.3	54.1 ± 3.6	16.9 ± 4.1
*R* ^2^	1.000	.999	.999	.997	.921	.976	.997	.997	.999	.999	.999

### 3.4 Oxidative stability

**Figure 7:**
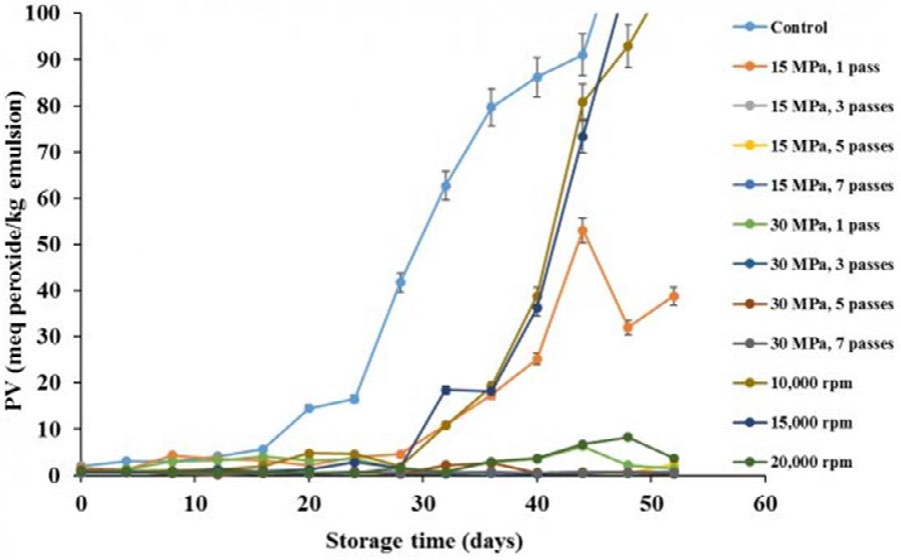
Change in the peroxide value of camelina seed oil emulsions prepared using high‐pressure and high shear homogenizer during accelerated oxidation test at 50°C in open‐lid containers

Figure 7 presents the peroxide formation in the camelina seed oil emulsions during accelerated oxidation test. During the early storage periods (16 days), there was no significant difference in the peroxide value of the emulsions compared to control camelina seed oil (*p* > .05); however, control sample started to oxidize on day 16 while there was no significant peroxide formation in all emulsions until day 28. The emulsions formed using the high shear homogenizer at 10,000 and 15,000 rpm and the high‐pressure homogenizer at 15 MPa with 1 pass presented a significant increase in their peroxide values on day 28 by reaching peroxide values above 50 meq/kg, whereas peroxide value increased for the ones prepared at 20,000 rpm shear rate and 30 MPa with 1 pass on the 32^nd^ day and reached their maximum value around 10 meq peroxide/kg. Emulsions prepared with high‐pressure homogenization at both 15 and 30 MPa with 3 and more passes did not exhibit any peroxide formation during the entire test. These results showed that the emulsions that were physically stable were more stable against oxidation. Kuhn and Cunha et al. (2012) reported similar observations except for the flaxseed oil emulsion prepared at 80 MPa which showed a significant increase in peroxide value, which could be due to excessive heat generated by high pressure. Oxidative stability of the more physically stable camelina seed oil emulsions was partly attributed to the physical protection provided by WPI. The dispersed oil droplets were covered by WPI and therefore protected from oxygen. A similar explanation for the protection of flaxseed oil from oxidation by different protein emulsifiers was reported by Karaca, Nickerson, and Low (2013).

## 4 CONCLUSIONS

Stable camelina seed oil emulsions emulsified by WPI were successfully formed using high‐pressure homogenizers. Higher pressures (30 MPa) and number of passes (>3) resulted in higher physical and oxidative stability of the emulsions. Oxidative stability of camelina seed oil emulsions was higher than that of control camelina seed oil. Emulsions which were physically stable were more stable against oxidation. Increasing pressure resulted in reduction in particle size where the lowest average particle size (278 nm) was obtained at 30 MPa with 3 number of passes. The use of high shear homogenizer, however, resulted in particle sizes which are eight‐fold (~2,517 nm) higher than the values obtained using high shear homogenizer. In addition, all camelina seed oil emulsions exhibited a shear thinning behavior. High‐pressure homogenization at 30 MPa with 5 passes was found to be the best emulsion formation condition due to higher oxidative stability and physical stability.

## 5 CONFLICT OF INTEREST

The authors declare no conflict of interest.
